# Supplementary material for: Exercise facilitates post-stroke recovery through mitigation of neuronal hyperexcitability via interleukin-10 signaling
Source: Nat Commun. 2025 Oct 8;16:8928. doi: 10.1038/s41467-025-62631-y (PMC12508215; doi:10.1038/s41467-025-62631-y)
Supplement: Supplementary file 1 — Supplementary Information [file 41467_2025_62631_MOESM1_ESM.pdf]

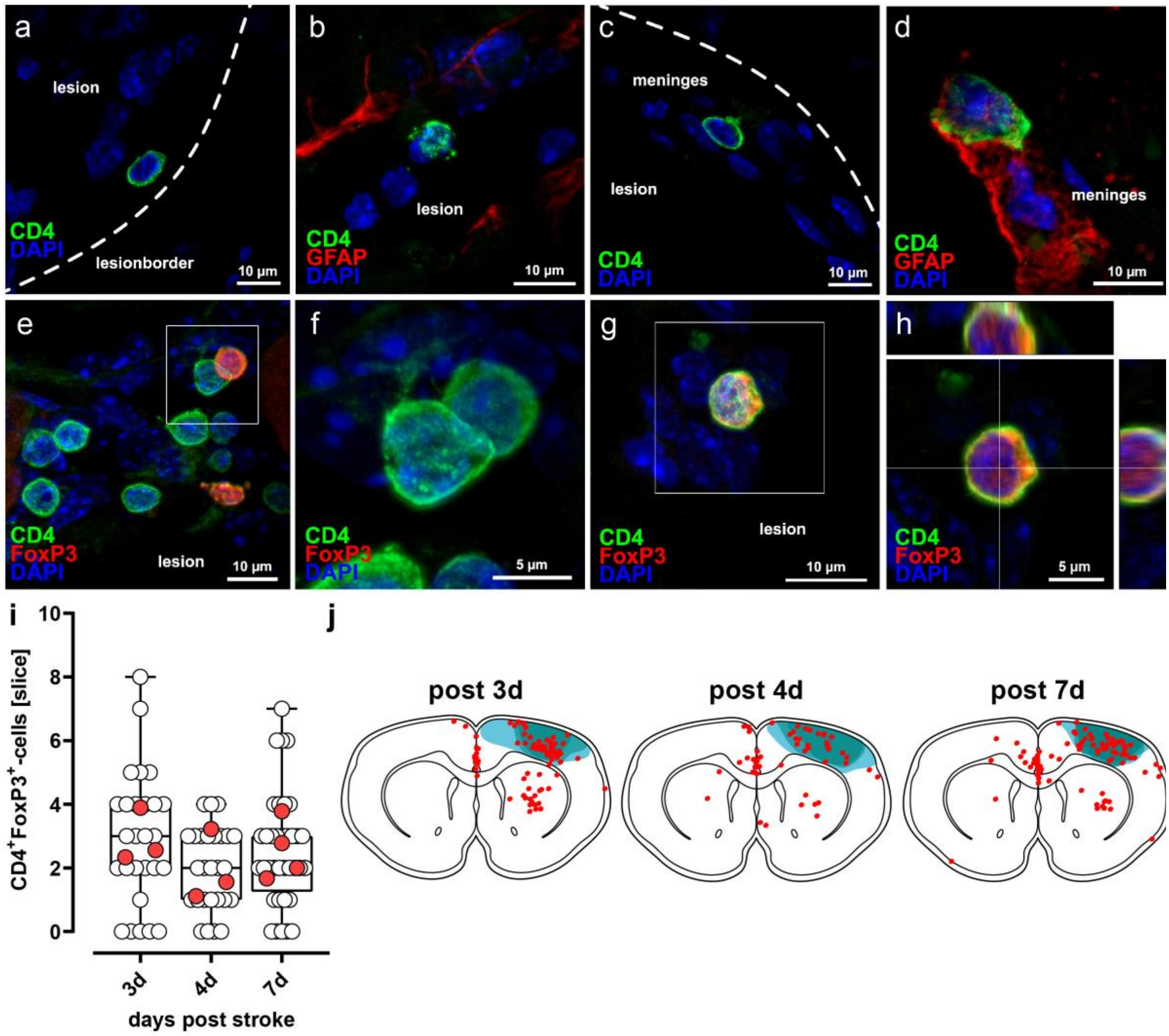

Supplementary Figure 1: Adoptively transferred T cells migrate into the ischemic hemisphere of RAG-mice subjected to experimental stroke. **a, b** CD4$^+$-T cells adoptively transferred to mice subjected to experimental stroke within the ischemic parenchyma. **c** Administered CD4$^+$-T cells within the ipsilateral meninges. **d** Double staining of CD4$^+$-T cell and Gfap$^+$-astrocyte within the ipsilateral meninges. **e** Immunofluorescence image of CD4$^+$-T cells and expression of Treg-reporter FoxP3-RFP within the ischemic parenchyma shows a CD4$^+$FoxP3$^-$- and CD4$^+$FoxP3$^+$-T cell in close contact. **f** Magnification indicates that both T cells in e. express the T cell marker CD4. **g** Maximum intensity plot of a CD4$^+$FoxP3$^+$-Treg within the ischemic parenchyma. Panels a-g display representative images; similar results were observed in all animals (n = 3 per time point). **h** Magnification of z stack shows FoxP3-enriched protrusions close to the CD4$^+$-membrane. Nuclear counterstain with 4',6-diamidino-2-phenylindole (DAPI). **i** Time-course analysis of adoptively transferred CD4$^+$FoxP3-RFP$^+$-

Treg infiltration into the ischemic hemisphere of RAG-1$^{-/-}$-mice 3-, 4- and 7-days post stroke (each time-point n = 3 animals, white dots show individual measurements of all sampled slices; red dots show the mean for each mouse). **j** Heatmaps depicting the location of immigrated adoptively transferred Cd4^{+}FoxP3-RFP^{+}-Tregs relative to the infarct within the brains of RAG-mice subjected to experimental stroke. Each red dot represents one Treg-cell. Stacked turquoise areas mark the infarcted tissue of each animal (per time-point n = 3 animals). Source data are provided as a Source Data file.

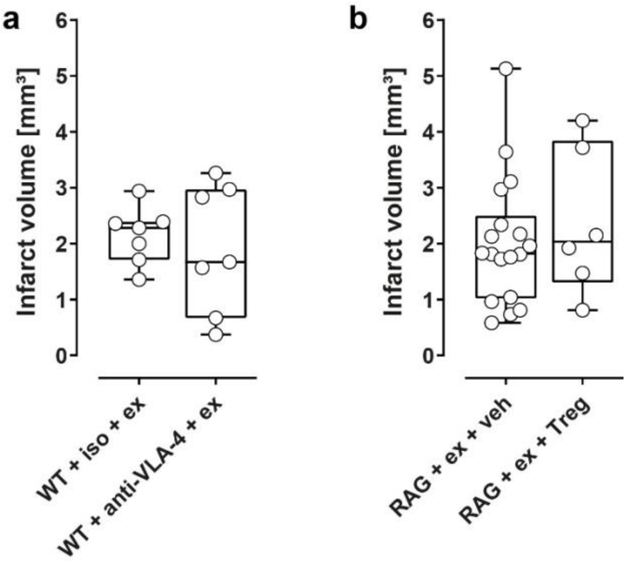

Supplementary Figure 2. Effects of VLA-4-blockade and Treg-transfer on infarct volumes. a Infarct volumes of wild-type (WT) mice 24 hours after stroke treated either with isotype control antibody or anti-VLA-4 antibody. No significant differences in infarct size were observed between groups (isotype: 2.14 ± 0.19 mm³ vs. anti-VLA-4: 1.90 ± 0.43 mm³; p = 0.60, two-sided t-test; n = 7 animals each). **b** Infarct volumes of RAG-1$^{-/-}$-mice 24 hours after stroke receiving either vehicle transfer and exercise or Treg transfer and exercise. Again, no significant differences in infarct size were detected (vehicle + exercise: 2.03 ± 0.27 mm³ vs. Treg transfer + exercise: 2.38 ± 0.54 mm³; p = 0.54, two-sided t-test; n = 18 and 6 animals, respectively. For box and whisker plots, the box extends from the 25th to the 75th percentile, the centre is the median and whiskers extend from the minimum or maximum. Overlaid dots represent individual values for each animal. Each dot represents an individual biological replicate. Source data are provided as a Source Data file.

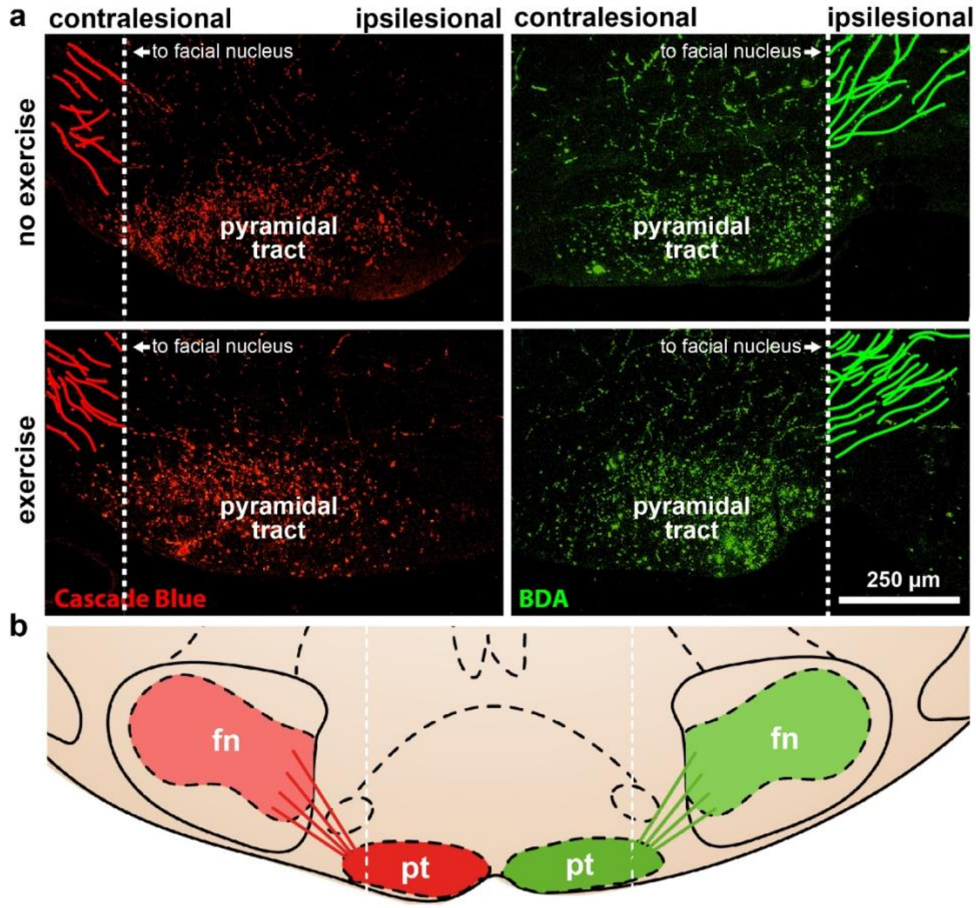

Supplementary Figure 3: Tract tracing quantification a Tracer marked sprouting tracts starting from pyramidal tracts towards facial nucleus were quantified from a predefined intersection (dotted white line, counted tracts marked in green for BDA and red for Cascade blue). **b** Schematic diagram showing the quantified tracer marked projections to the facial nucleus.

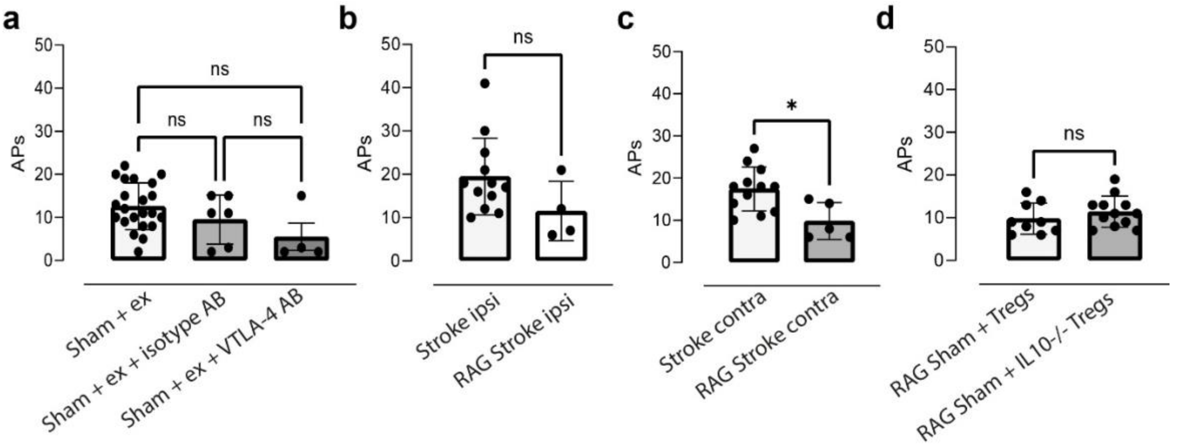

Supplementary Figure 4: Control conditions strengthen the specific impact of exercise on altered cortical excitability in stroke.

a Blockade of leukocyte–endothelial interaction with isotype control antibody (AB), or with VLA-4 AB treatment, does not alter cortical neuron excitability in WT sham animals undergoing exercise ($p > 0.05$, Kruskal–Wallis test + Dunn's correction, n = 4-22*).* **b, c** Comparison of AP counts between stroke WT and stroke RAG-$1^{-/-}$-mice shows no significant difference in excitability on the ipsilateral side (**b**, p = 0.13, two-sided t-test, n = 4-12 animals per group), but reveals a significant genotype effect on the contralateral side (**c***,* *p = 0.012, two-sided t-test, n = 5-12 animals per group). **d** IL-$10^{-/-}$ Tregs do not further exacerbate hyperexcitability in RAG sham animals without exercise (p = 0.32, two-sided t-test, n = 9-12 animals per group*).* Each dot represents an individual biological replicate. Source data are provided as a Source Data file.

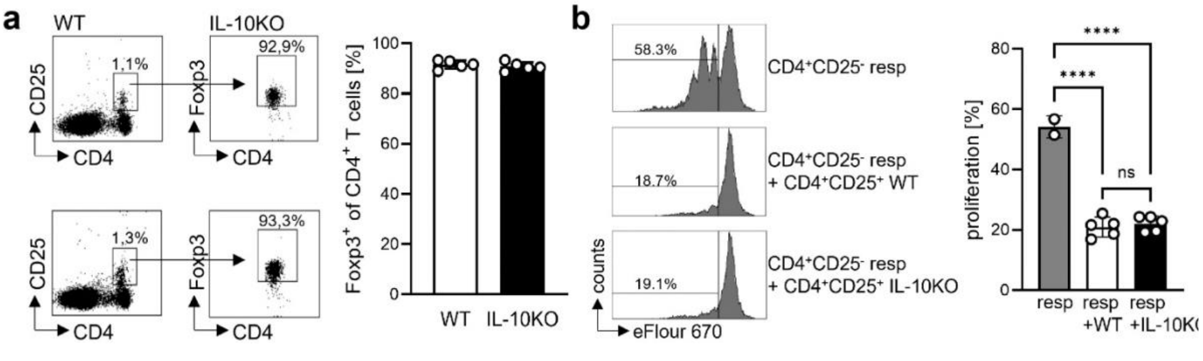

Supplementary Figure 5: IL-10 deficiency has no impact on FoxP3 expression and inhibitory activity of Tregs. a FoxP3 expression in gated $CD4^{+}CD25^{+}$ Tregs from IL-10 deficient (IL-10KO) and C57BL/6 (WT) mice analyzed by flow cytometry. **b** Proliferation of $CD4^{+}CD25^{-}$ responder cells (resp) stimulated with anti-CD3 and cultured alone or co-cultured with $CD4^{+}CD25^{+}$ Tregs from IL-10 deficient (IL-10KO) and C57BL/6 (WT) mice at a ratio 1:1 was analyzed by flow cytometry. Results are summarized as mean +/- SD from n = 2 – 5 animals. ****p<0.001, One-Way ANOVA. Source data are provided as a Source Data file.

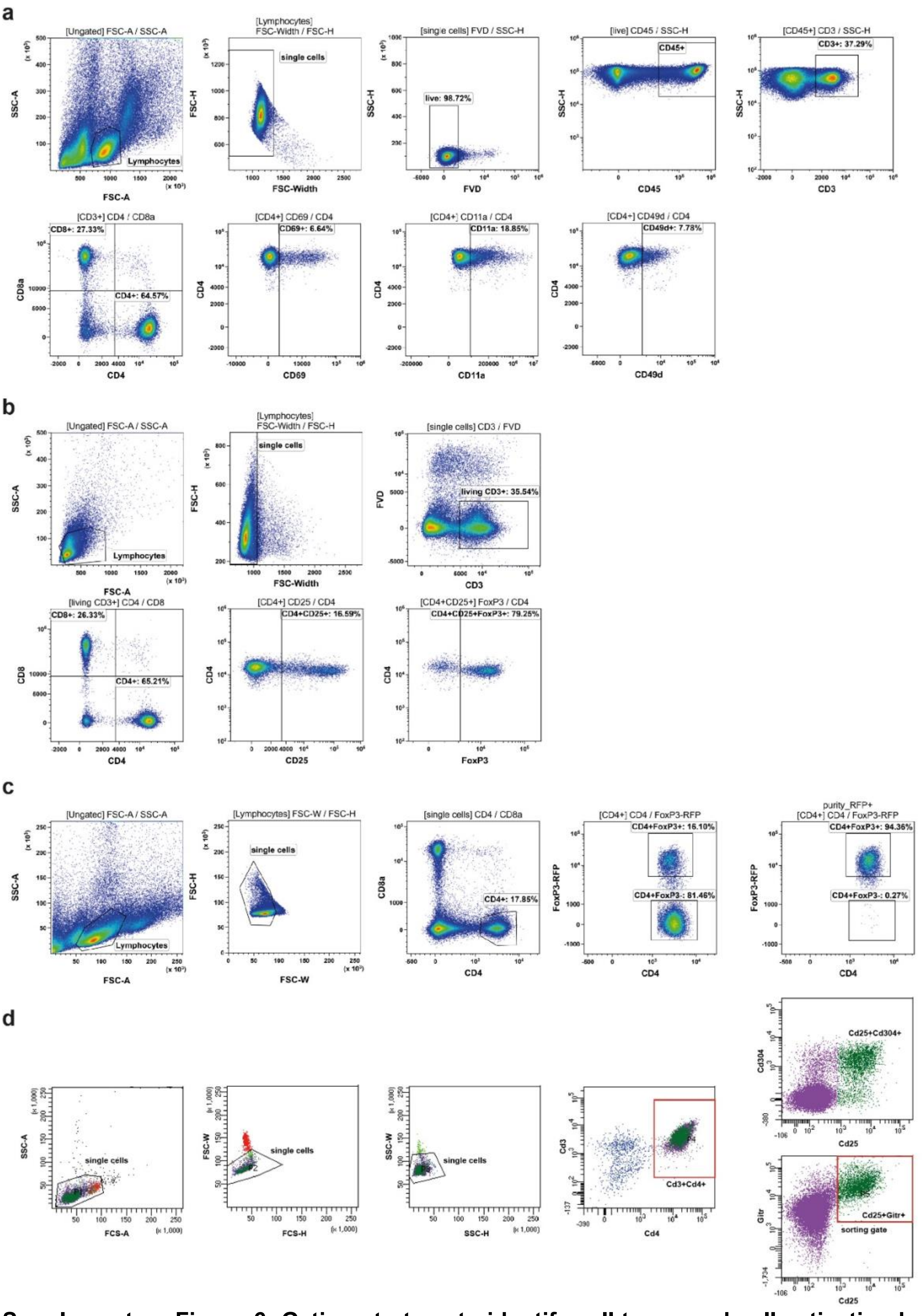

Supplementary Figure 6: Gating strategy to identify cell types and cell activation by flow cytometry. A Representative gating strategy for characterization and quantification of CD4^{+}-T cell activation and migration markers CD69, CD11a and CD49a. **B** Representative gating strategy for identification and quantification of CD4^{+}Foxp3^{+}-regulatory T cells. **C**

Gating strategy for sorting of CD4$^+$Foxp3-RFP$^+$-regulatory T cells in FoxP3-RFP-reporter mice. **D** Gating strategy for sorting of CD4$^+$Cd25$^+$-regulatory T cells in non-reporter mice. Markers CD304 and Gitr served as additional quality control.
