# Supplementary material for: Exercise facilitates post-stroke recovery through mitigation of neuronal hyperexcitability via interleukin-10 signaling
Source: Nat Commun. 2025 Oct 8;16:8928. doi: 10.1038/s41467-025-62631-y (PMC12508215; doi:10.1038/s41467-025-62631-y)
Supplement: Supplementary file 2 — Reporting Summary [file 41467_2025_62631_MOESM2_ESM.pdf]

nature portfolio

Corresponding author(s): Jens Minnerup

Last updated by author(s): Jul 7, 2025

Reporting Summary

Nature Portfolio wishes to improve the reproducibility of the work that we publish. This form provides structure for consistency and transparency in reporting. For further information on Nature Portfolio policies, see our Editorial Policies and the Editorial Policy Checklist.

Statistics

For all statistical analyses, confirm that the following items are present in the figure legend, table legend, main text, or Methods section.

n/a	Confirmed	
☐	☒	The exact sample size (*n*) for each experimental group/condition, given as a discrete number and unit of measurement
☐	☒	A statement on whether measurements were taken from distinct samples or whether the same sample was measured repeatedly
☐	☒	The statistical test(s) used AND whether they are one- or two-sided<br>*Only common tests should be described solely by name; describe more complex techniques in the Methods section.*
☐	☒	A description of all covariates tested
☐	☒	A description of any assumptions or corrections, such as tests of normality and adjustment for multiple comparisons
☐	☒	A full description of the statistical parameters including central tendency (e.g. means) or other basic estimates (e.g. regression coefficient) AND variation (e.g. standard deviation) or associated estimates of uncertainty (e.g. confidence intervals)
☐	☒	For null hypothesis testing, the test statistic (e.g. F, t, r) with confidence intervals, effect sizes, degrees of freedom and P value noted<br>*Give P values as exact values whenever suitable.*
☒	☐	For Bayesian analysis, information on the choice of priors and Markov chain Monte Carlo settings
☒	☐	For hierarchical and complex designs, identification of the appropriate level for tests and full reporting of outcomes
☐	☒	Estimates of effect sizes (e.g. Cohen's *d*, Pearson's *r*), indicating how they were calculated

Our web collection on statistics for biologists contains articles on many of the points above.

Software and code

Policy information about availability of computer code

Data collection	N/A
Data analysis	We used GraphPad Prism version 10 (GraphPad Software, La Jolla, CA) for statistical analyses.

For manuscripts utilizing custom algorithms or software that are central to the research but not yet described in published literature, software must be made available to editors and reviewers. We strongly encourage code deposition in a community repository (e.g. GitHub). See the Nature Portfolio guidelines for submitting code & software for further information.

Data

Policy information about availability of data

All manuscripts must include a data availability statement. This statement should provide the following information, where applicable:

- Accession codes, unique identifiers, or web links for publicly available datasets
- A description of any restrictions on data availability
- For clinical datasets or third party data, please ensure that the statement adheres to our policy

Source data underlying Figs. 1-7 and Supplementary Figs. 1, 2, 4 and 5 are provided in the source data file. Source data are provided with this paper.

Research involving human participants, their data, or biological material

Policy information about studies with human participants or human data. See also policy information about sex, gender (identity/presentation), and sexual orientation and race, ethnicity and racism.

Reporting on sex and gender	N/A
Reporting on race, ethnicity, or other socially relevant groupings	N/A
Population characteristics	N/A
Recruitment	N/A
Ethics oversight	N/A

Note that full information on the approval of the study protocol must also be provided in the manuscript.

Field-specific reporting

Please select the one below that is the best fit for your research. If you are not sure, read the appropriate sections before making your selection.

- [x] Life sciences
- [ ] Behavioural & social sciences
- [ ] Ecological, evolutionary & environmental sciences

For a reference copy of the document with all sections, see nature.com/documents/nr-reporting-summary-flat.pdf

Life sciences study design

All studies must disclose on these points even when the disclosure is negative.

Sample size	A priori sample size calculations were performed to achieve 80% power to detect a relevant treatment effect of 25 % with an alpha level of 0.05. We used the sample size calculator available at http://www.stat.ubc.ca.
Data exclusions	During the experiment, animals were excluded if, one day after ischemia induction, they either exhibited no stroke-related deficits or were severely affected to the extent that they would be unable to commence treadmill training two days after ischemia induction. These exclusion criteria were predetermined.
Replication	Our eperiments were all performed in more than just one individual animals. The given n for each experiment reflects the number of individual animals. Each dot represents an individual biological replicate.
Randomization	The randomization process was conducted using Research Randomizer (Version 4.0), developed by Urbaniak, G. C., & Plous, S. (2013).
Blinding	Functional testing, MRI measurements, quantification of histological findings and analyses of neuronal activity were performed in a blinded manner.

Reporting for specific materials, systems and methods

We require information from authors about some types of materials, experimental systems and methods used in many studies. Here, indicate whether each material, system or method listed is relevant to your study. If you are not sure if a list item applies to your research, read the appropriate section before selecting a response.

Materials & experimental systems

n/a	Involved in the study	
[ ]	[x]	Antibodies
[x]	[ ]	Eukaryotic cell lines
[x]	[ ]	Palaeontology and archaeology
[ ]	[x]	Animals and other organisms
[x]	[ ]	Clinical data
[x]	[ ]	Dual use research of concern
[x]	[ ]	Plants

Methods

n/a	Involved in the study	
[x]	[ ]	ChIP-seq
[ ]	[x]	Flow cytometry
[ ]	[x]	MRI-based neuroimaging

Antibodies

Antibodies used	We used the following primary antibodies for murine sections: Rabbit-anti-Arginase-1 (1:100, Abcam, ab91279), Hamster-anti-CD3 (1:50, BD Bioscience, 550277), Goat-anti-CD206 (1:100, R&D Systems, AF2535), Rat-anti-F4/80 (1:500, clone CI:A3-1, Serotec,

MCA497G), Mouse-anti-GFAP (1:500, clone G-A-5, Millipore, G3893), Goat-anti-Iba1 (1:50, Abcam, ab5076), Rat-anti-Ly-6B.2 (1:100, clone 7/4, BioRad, MCA771G), Chicken-anti-MAP-2 (1:100, Abcam, ab5392), Rabbit-anti-NeuN (1:150, clone 27-4, Millipore, MABN140).
For flow cytometry, we used the following fluorochrome-labeled antibodies: CD45 (30-11F) BV510 or FITC 1:100, CD45R/B220 (RA3-6B2) PerCP-Cy5.5 1:100, CD3 (17A2) PE-Cy7 1:200, F4/80 (BM8) APC 1:200, Ly-6G/Ly-6C (RB6-8C5) BV421 1:200, CD11c (N418) AF700 1:150, CD11b (M1/70) PE 1:800 all obtained from Biolegend and NK-1.1 and (PK136) APC-Vio770 1:200 obtained from Miltenyi Biotec, and CD3 (145-2C11) 1:200, CD4-Pacific Blue (RM4-4) 1:150, CD8a-FITC (53-6.7) 1:150, CD25-APC (PC61) 1:150, Ki-67 AF647 (B56) 1:200 from BD Biosciences, and FoxP3 (FJK-16s) PE 1:150 from eBioscience

Validation

To confirm specificity, the antibody protocol was carried out in parallel without the primary antibody as a negative control. These control experiments showed no detectable signal, confirming the specificity of the staining.

Animals and other research organisms

Policy information about studies involving animals; ARRIVE guidelines recommended for reporting animal research, and Sex and Gender in Research

Laboratory animals

We used solely adult and male mice, in detail C57BL/6 mice, RAG-1-deficient C57BL/6 mice, FoxP3-mRFP C57BL/6 mice (C57BL/6-Foxp3tm1Flv/J), scurfy mice on a C57BL/6 background (B6.Cg-Foxp3sf/Y) and IL-10-deficient C57BL/6 mice. All mice were 11-13 weeks of age, except for scurfy mice (2-3 weeks of age) and IL-10-deficient C57BL/6 mice (10-12 weeks of age). Mice were kept under standard housing conditions with a 12:12 h light-dark cycle, an ambient temperature between 20–24 °C and humidity between 45–65%. Mice had free access to pelleted food and water. To minimize potential confounders, animals from different treatment groups were housed together in the same cage, and all treatments and measurements were conducted at the same time of day under identical conditions.
Experiments were performed in accordance with the ARRIVE guidelines (the checklist is provided in the supplement).
We used solely adult and male mice, in detail C57BL/6 mice, RAG-1-deficient C57BL/6 mice, FoxP3-mRFP C57BL/6 mice (C57BL/6-Foxp3tm1Flv/J), scurfy mice on a C57BL/6 background (B6.Cg-Foxp3sf/Y) and IL-10-deficient C57BL/6 mice. All mice were 11-13 weeks of age, except for scurfy mice (2-3 weeks of age) and IL-10-deficient C57BL/6 mice (10-12 weeks of age). Mice were kept under standard housing conditions with a 12:12 h light-dark cycle, an ambient temperature between 20–24 °C and humidity between 45–65%. Mice had free access to pelleted food and water. To minimize potential confounders, animals from different treatment groups were housed together in the same cage, and all treatments and measurements were conducted at the same time of day under identical conditions.
Experiments were performed in accordance with the ARRIVE guidelines (the checklist is provided in the supplement).

Wild animals

N/A

Reporting on sex

We used solely male mice

Field-collected samples

N/A

Ethics oversight

All animal procedures were performed in accordance with local animal welfare regulations and experimental protocols were approved by the local governmental authorities (Landesamt für Natur, Umwelt und Verbraucherschutz, NRW, Germany) under the approval reference number (AZ 84-02.04.2015.A476).

Note that full information on the approval of the study protocol must also be provided in the manuscript.

Plants

Seed stocks

N/A

Novel plant genotypes

N/A

Authentication

N/A

Flow Cytometry

Plots

Confirm that:

- [x] The axis labels state the marker and fluorochrome used (e.g. CD4-FITC).
- [x] The axis scales are clearly visible. Include numbers along axes only for bottom left plot of group (a 'group' is an analysis of identical markers).
- [x] All plots are contour plots with outliers or pseudocolor plots.
- [x] A numerical value for number of cells or percentage (with statistics) is provided.

Methodology

Sample preparation	After staining, cells were washed twice and resuspended in PBS with 2% FCS. Cells were acquired on a Gallios flow cytometer (Beckman Coulter) or FASC Symphony (BD Biosciences) or sorted on a FACS Aria III (BD). Sorting was performed using an 85 µm nozzle and 4-way purity sort precision mode. Data were analyzed using FlowJo software v10.6.1 (BD). Cell concentrations from all tissues were manually counted in a Fuchs-Rosenthal counting chamber.
Instrument	Cells were acquired on a Gallios flow cytometer (Beckman Coulter) or FASC Symphony (BD Biosciences) or sorted on a FACS Aria III (BD).
Software	Data were analyzed using FlowJo software v10.6.1 (BD). Cell concentrations from all tissues were manually counted in a Fuchs-Rosenthal counting chamber.
Cell population abundance	Sorting was performed using an 85 µm nozzle and 4-way purity sort precision mode.
Gating strategy	N/A

- [ ] Tick this box to confirm that a figure exemplifying the gating strategy is provided in the Supplementary Information.

Magnetic resonance imaging

Experimental design

Design type	N/A
Design specifications	N/A
Behavioral performance measures	N/A

Acquisition

Imaging type(s)	Whole skull ex vivo diffusion-weighted magnetic resonance imaging (dMRI)
Field strength	9.4 Tesla
Sequence & imaging parameters	MR images were acquired on a 9.4 T horizontal animal scanner with 20 cm bore (94/20 USR BioSpec, Avance III, Bruker BioSpin, Ettlingen, Germany), equipped with a 700 mT/m gradient system (BGA12S, BrukerBioSpin) and a 2-element cryogenic transceiver coil (Bruker BioSpin) for image acquisition. The system is operated by ParaVision 6.0.1 (Bruker BioSpin). Images were acquired with a multishot 3D spin-echo EPI sequence with the following scan parameters: echo time = 24.5 ms, repetition time = 300 ms, 8 segments, bandwidth = 250 kHz, 100x100x100 µm³ resolution, gradient duration = 4.5 ms, gradient separation = 11 ms, 80 diffusion directions, b-values = 1500 and 3000 s/mm².
Area of acquisition	Whole skull

Diffusion MRI [x] Used [ ] Not used

Parameters: 80 diffusion directions, b-values = 1500 and 3000 s/mm².

Preprocessing

Preprocessing software	Whole skulls were embedded in 1% low-melting agarose (Sigma Aldrich, St. Louis, USA) with 2 mM Magnevist. MR images were acquired on a 9.4 T horizontal animal scanner with 20 cm bore (94/20 USR BioSpec, Avance III, Bruker BioSpin, Ettlingen, Germany), equipped with a 700 mT/m gradient system (BGA12S, BrukerBioSpin) and a 2-element cryogenic transceiver coil (Bruker BioSpin) for image acquisition. The system is operated by ParaVision 6.0.1 (Bruker BioSpin). Fitting of diffusion metrics and fiber tracking were performed using DSI Studio (http://dsi-studio.labsolver.org/). The restricted diffusion was quantified using restricted diffusion imaging36. The diffusion data were reconstructed using generalized q-sampling imaging37 with a diffusion sampling length ratio of 1.4.

Normalization	N/A
Normalization template	N/A
Noise and artifact removal	N/A (post mortem MRI)
Volume censoring	N/A

Statistical modeling & inference

Model type and settings	*Specify type (mass univariate, multivariate, RSA, predictive, etc.) and describe essential details of the model at the first and second levels (e.g. fixed, random or mixed effects; drift or auto-correlation).*
Effect(s) tested	*Define precise effect in terms of the task or stimulus conditions instead of psychological concepts and indicate whether ANOVA or factorial designs were used.*

Specify type of analysis: ☐ Whole brain ☐ ROI-based ☒ Both

Anatomical location(s)	Lesion volumetry was performed on the dMRI scans from a manual outline of cortical voxels with signal voids or aberrant anisotropy vectors. To quantify interhemispheric connectivity, we used a deterministic fiber tracking algorithm as previously described38. The midline of the corpus callosum was chosen as seed region (delineated by dominant left-right diffusion anisotropy, 50000 seeds). The anisotropy threshold was 0.03, angular threshold 60°. The step size was 0.03 mm. Tracks with length shorter than 1 or longer than 20 mm were discarded.
Statistic type for inference (See Eklund et al. 2016)	N/A
Correction	N/A

Models & analysis

n/a	Involved in the study
☒	☐ Functional and/or effective connectivity
☒	☐ Graph analysis
☒	☐ Multivariate modeling or predictive analysis
